# Museum-based activities and psychological wellbeing: a pre–post study of visual thinking strategies and guided visits across two cultural settings

**DOI:** 10.3389/fpsyg.2026.1904352

**Published:** 2026-10-19

**Authors:** Claudio Lucchiari, Maria Elide Vanutelli, Vincenza Ferrara, Annalisa Banzi

**Affiliations:** 1 Department of Philosophy “Piero Martinetti”, Università degli Studi di Milano, Milan, Italy; 2 Department of Psychology, University of Milan-Bicocca, Milan, Italy; 3 Sapienza University of Rome, Rome, Italy

**Keywords:** ASBA project, content analysis, cultural heritage, stress, museum, wellbeing, personality traits, state anxiety, visual thinking strategies

## Abstract

**Introduction:**

Museum visits are increasingly recognised as contributors to psychological well-being, but less is known about whether museum activities designed for educational purposes are associated with short-term affective changes. Within the ASBA (Anxiety, Stress, Brain-friendly museum, Approach) project, this study examined two educational (non-therapeutic) activities, a visual thinking strategies (VTS) session and a standard guided visit, conducted in two culturally distinct settings in Milan, Italy: the Modern Art Gallery (MAG) and the Natural History Museum (NHM).

**Methods:**

In a pre–post design with two between-subjects factors (activity and museum), 135 adults attending 11 group sessions (VTS: *n*= 85, 7 sessions; guided visit: *n*= 50, 4 sessions) completed the State Anxiety Inventory (SAI) and six Visual Analogue Scales (VAS) before and after the activity and briefly described their experience in an open-ended question. State anxiety was the primary, confirmatory outcome; all other analyses were exploratory. Changes were analysed with multilevel models including a random intercept for session, and the open-ended responses with a descriptive content analysis.

**Results:**

State anxiety decreased from pre to post (*Δ* = −5.55, 95% CI [−6.85, −4.26]; d_av = −0.81) and mood improved (*Δ* = +1.07, 95% CI [0.74, 1.40]; d_av = 0.72), with negligible dependence within sessions (ICC ≤ 0.04). In a multilevel ANCOVA adjusting for baseline anxiety, no statistically detectable difference emerged between activities (adjusted difference = 0.38 SAI points, 95% CI [−2.67, 3.42]). The content analysis suggested that participants reached these changes through different experiential routes: cognitive descriptions prevailed after VTS, emotional-aesthetic descriptions after the guided visit, and social-relational descriptions were reported only after VTS.

**Discussion:**

Both educational activities were followed by comparable short-term improvements in state anxiety and mood, although participants described them in different terms. Because the study did not include a non-museum control condition and allocation was not randomised, these changes cannot be causally attributed to the museum activities. The findings are preliminary and hypothesis-generating and warrant controlled studies of museum-based activities for wellbeing.

## 1 Introduction

### 1.1 Cultural heritage as a tool for life skills empowerment

Cultural heritage is not only a valuable repository of historical, artistic, and scientific knowledge but also a significant resource for promoting learning, supporting social inclusion, and enhancing wellbeing, including within therapeutic and health-related contexts (Cardillo and Chatterjee, 2025; Dow et al., 2023; Cotter and Pawelski, 2021; Fudickar et al., 2022). As embodied in objects encountered in museums and other cultural institutions, cultural heritage lends itself to multiple and transdisciplinary interpretations, further enhanced by digital reproduction processes that can improve accessibility, visitor engagement, and visitor wellbeing (Cotter et al., 2024).

Museum objects may be analysed and contextualised through diverse interpretative models, including acquisition records; the cultural and scientific milieu of their production or development; documentation relating to conservation and restoration; their function as research data within scholarly inquiry; their placement within the museum itinerary (chronological, thematic, etc.); their material composition and intended function; and their representational status as images associated with specific concepts. In addition, museum objects may elicit differentiated affective and cognitive responses, which vary according to visitors’ age, prior experience, and level of expertise.

These characteristics support the embedding of cultural heritage into educational models oriented towards the development of the Life Skills recommended by WHO (1999), conceived as essential competencies for addressing the demands, pressures, and stressors of everyday life and for advancing overall quality of life. Life Skills may be categorised into three principal domains: emotional, which includes self-awareness, emotion regulation, and stress management; relational, which includes empathy, effective communication, and constructive interpersonal relationships; and cognitive, which is more related to problem solving, decision making, critical thinking, and creative thinking.

The effective implementation of such models requires active, participatory educational approaches that position learners as agents in their own developmental processes, thereby fostering the identification of adjustable strategies and supportive contexts conducive to personal wellbeing. Within this framework, museum-based cultural heritage has been increasingly recognised not only as an educational asset but also as a contributory factor to psychological and physiological wellbeing (Chatterjee and Camic, 2015; Desmarais et al., 2018; Golden et al., 2024; Jensen and Bonde, 2018). Empirical evidence indicates that engagement with the arts is associated with stress reduction (Grossi et al., 2019; Vanutelli et al., 2025b); enhanced self-reflection and self-awareness and modifications in behavioural patterns (Yifei and Nasri, 2025); normalisation of heart rate, blood pressure, and cortisol levels (Bolwerk et al., 2014); and contributions to motor neurorehabilitation (Iosa et al., 2022), among other documented outcomes.

### 1.2 The ASBA project

The ASBA Project (Anxiety, Stress, Brain-friendly museum, Approach; Banzi, 2023) is positioned within this framework and entails collaboration among university researchers, museum staff, and experts in different methods applied in selected museums. It is based on a rigorous research protocol (Lucchiari et al., 2024) that uses validated and standardised questionnaires, BCI (Brain-Computer Interface), and focus groups to assess participants’ anxiety and stress levels and promote wellbeing.

The project is divided into three distinct phases to enhance the wellbeing of adult citizens (Banzi et al., 2026), museum personnel (Vanutelli et al., 2025a), and minors.

The ASBA project pursues several objectives, including enhancing the wellbeing of both citizens and museum staff. This aim aligns with the expanding field of Arts, Health, and Wellbeing, and recognises museums as environments with regenerative potential. The project is structured to gather evidence supporting this capacity for both visitors and cultural sector employees. This primary objective is integrated with efforts to valorise and promote museums and their collections. The project methodology encourages participants to slow down, observe objects attentively, and engage in critical, shared interpretation. This approach moves beyond standard factual label reading, fostering a deeper and more meaningful personal connection between visitors and the collection.

### 1.3 The role of visual thinking strategies

A variety of activities can be implemented within museums to promote focus and wellbeing. The ASBA protocol includes mindfulness, art therapy, the Artup method, the Nature + Art method, chair yoga, and visual thinking strategies (VTS).

The VTS method was developed in the United States beginning in 1988 through the collaboration between Abigail Housen (a Harvard cognitive psychologist and researcher) (Housen, 2002) and Philip Yenawine (Director of Educational Activities at MOMA in New York) (Yenawine, 2013). Grounded in cognitive developmental theory and museum education practice, the method is designed to foster structured, facilitated discussions around works of art. In practice, a VTS session is a group discussion led by a trained facilitator: participants first observe an image in silence for a short time and then respond to three open-ended questions (“What’s going on in this picture?”, “What do you see that makes you say that?”, and “What more can we find?”). The facilitator does not provide information about the work during the discussion, but paraphrases each contribution, points to the details mentioned, and links different viewpoints, thereby supporting a collective, evidence-based construction of meaning (Yenawine, 2013).

Among the outcomes attributed to VTS, those most relevant to the present study concern observational skills, the capacity to tolerate ambiguity and consider multiple perspectives, and the quality of dialogical exchange within the group, including active listening and respect for differing viewpoints (Housen, 2002; Yenawine, 2013). VTS has been successfully applied in medical education as well as other professional fields (Klugman et al., 2011; Poirier et al., 2020; see also Cerqueira et al., 2023).

These outcomes are closely related to the methodological structure of VTS, which situates reasoning within a social framework by integrating cognitive processes with social interaction. This approach reflects theoretical perspectives advanced by Piaget (2000), Dewey (1934), Montessori (1909), and Vygotskij (1934), particularly regarding the interplay between individual cognitive development and socially mediated learning.

Beyond these educational outcomes, VTS has been associated with psychological and neurofunctional effects. Neuroscientific work has explored the mechanisms underlying the VTS process, identifying patterns of activation associated with the social brain and suggesting how structured aesthetic dialogue may influence socio-cognitive functioning (van Leeuwen et al., 2023).

Empirical studies have also evaluated VTS as a strategy for promoting wellbeing. Studies incorporating repeated VTS sessions have reported reductions in burnout among healthcare workers (Ferrara et al., 2022a) and in symptoms of anxiety and depression among patients with systemic lupus erythematosus (Ferrara et al., 2022b).

In some of these studies, participants were asked, through a questionnaire administered immediately following the VTS session, to describe their subjective experience during the activity. The majority of responses referred to states characterised by calmness, relaxation, ease, comfort, and enjoyment.

VTS was introduced in Italy in 2014, where it has been employed to use cultural heritage as a tool for learning and social inclusion, to experiment with innovative approaches to museum engagement, and to foster collaboration between teachers and museum educators, in line with the objective of promoting Life Skills through art-based practices (Ferrara, 2016). Its application has also extended to the health sector, including its integration into university curricula in Medicine and Nursing Sciences (Ferrara, 2020).

### 1.4 The present study

Although the benefits of museum engagement for wellbeing are increasingly documented, less is known about whether museum activities designed for educational purposes are also associated with short-term affective changes, and whether such changes depend on the format of the activity or on the type of collection. VTS, in particular, has mainly been evaluated in educational and healthcare settings, often through repeated sessions, whereas evidence on single sessions delivered to adult museum visitors, and on how they compare with conventional guided visits, remains limited. Within the scope and objectives of the ASBA project, the present study addressed this gap by comparing two museum activities: a VTS session, in which a trained facilitator leads a group discussion without providing information in advance, and a standard guided visit, in which a museum expert presents the collection through an expository tour (see Sections 2.4.4 and 2.4.5). It is important to note that both VTS and the standard guided visit were originally developed as educational strategies, not as interventions for anxiety or stress reduction. Accordingly, the present study did not hypothesise that VTS would outperform the standard visit. Rather, it examined whether activities designed for educational ends would nonetheless be associated with measurable short-term changes in state anxiety and mood, and whether such changes would be observed across different types of cultural heritage. The standard guided visit was included as a comparison condition representing typical museum practice, not as a control expected to be inferior. Activities were conducted at two distinct cultural institutions in Milan, Italy:

- The Modern Art Gallery (MAG), representing an art museum asset;
- The Natural History Museum (NHM) represents a scientific and natural history asset.

This parallel approach across diverse museum environments was designed to explore whether the changes associated with the VTS method are also observed beyond its original disciplinary context.

Accordingly, the study addressed three research questions. RQ1 (confirmatory): does state anxiety decrease from before to after each museum activity? RQ2 (exploratory): do changes in state anxiety and mood differ by activity format and museum setting, and are they associated with personality traits? RQ3 (exploratory):. how do participants describe their experience of the two formats? RQ3 motivated a descriptive content analysis of participants’ brief open-ended responses: pre–post measures indicate whether change occurs, but not what participants perceived as salient in the activity. Our working hypothesis was that different activities might be associated with similar outcomes through different experiential routes, predominantly cognitive, emotional-aesthetic, or social-relational.

## 2 Materials and methods

### 2.1 Design

The study was conducted in two museum settings: the Modern Art Gallery (MAG; Milan, Italy), which hosts collections of Italian Romantic paintings and sculptures, and the Natural History Museum (NHM; Milan, Italy), characterised by tropical nature dioramas depicting diverse flora and fauna.

A mixed design was adopted. The experiment included two between-subjects factors: the experimental condition (VTS vs. standard museum visit) and the museum setting (MAG vs. NHM). Time (pre vs. post) was the within-subjects factor. The design did not include a non-museum control condition. Participants were allocated to one of the groups according to the session date they chose, without knowing which activity would be delivered. Each session took place on a different date, always at the same time of day (2:30 p.m.), and included all participants attending on that date. In total, 11 sessions were conducted: 7 VTS sessions (3 at MAG and 4 at NHM) and 4 guided-visit sessions (2 at MAG and 2 at NHM). Sessions included 8 to 16 participants (*M* = 12.3). Each participant took part in only one session and, therefore, in only one condition.

### 2.2 Participants

We used an opportunistic sampling strategy. Participants were recruited through social media platforms, newsletters, and the websites of institutional partners. Each month, prior to the commencement of the sessions, a registration calendar was made publicly available via a dedicated website as new participation slots opened. Registration closed on the day preceding each session to allow verification of eligibility criteria and completeness of documentation. Through the project’s dedicated webpage, participants scheduled their attendance and accessed the informed consent form.

The study was conducted in accordance with the Declaration of Helsinki and received approval from the local Ethical Committee (University of Milano-Bicocca; protocol code: 733/2022). All participants provided written informed consent after receiving detailed information regarding the study procedures, objectives, and potential risks. The consent document also explicitly stated the participant’s right to withdraw at any stage and included information regarding data protection and privacy policies.

Following consent, participants completed an online screening questionnaire to collect demographic information and verify eligibility. Subsequently, they completed the trait psychological questionnaires (see Section 2.4.2).

Eligibility criteria were initially assessed through the online self-report screening instrument, and the procedure was discontinued whenever responses indicated non-eligibility. Only volunteers who met all criteria were admitted to the study. Inclusion criteria were: age ≥ 18 years; acceptance of participation conditions; and signed informed consent. Exclusion criteria included: insufficient language proficiency to understand instructions and interact with facilitators and peers; current diagnosis of a psychiatric or neurological disorder; uncorrected visual or auditory impairments; or any medical condition potentially interfering with participation in the proposed activities.

Only participants who successfully completed the screening procedure and reserved a session were admitted to the on-site museum activities. Upon arrival, a researcher conducted a brief individual interview to verify the accuracy of the information provided online and to confirm understanding and acceptance of the informed consent. Each study session had a limit of 20 participants, and the rate of registered people who did not attend the session for unexplained reasons ranged from 20 to 60%. The data collected about of people who did not attended the museum activity were not used and deleted.

The data reported here have not been previously published. Participants do not overlap with the samples of previous ASBA publications, which examined other activities (Banzi et al., 2026; Vanutelli et al., 2025a).

### 2.3 Procedure

The procedure comprised six consecutive phases (see Figure 1).

**Figure 1:**
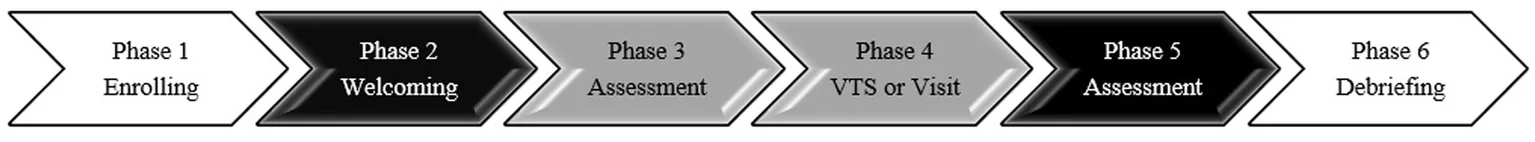
Graphic representation of the experimental procedure. The figure shows the six phases: (1) Enrolling and consent verification, (2) welcoming (3) pre-treatment assessment (SAI, VAS), (4) VTS or guided visit at MAG or NHM, (5) post-treatment assessment, (6) debriefing and information sharing.

*Phase 1*: Enrolling, informed consent and trait measurements (see Paragraph 2.2).

*Phase 2*: Welcoming. On the day of the session, participants were received in a designated museum area, where a check-in procedure confirmed completion of questionnaires and consent forms. Once all attendees had arrived, they were escorted to a room reserved for the study. Researchers reiterated essential study information, introduced the instructors and research staff, and provided a concise overview of the technique.

*Phase 3*: Pre-Treatment Assessment. Pre-intervention questionnaires were administered to assess state anxiety (SAI) and other psychological dimensions (VAS). Participants were free to select seating within a conference room to ensure comfort and privacy.

*Phase 4*: Museum activity. Depending on the assigned condition, participants took part either in the VTS intervention (see paragraph 2.4.4) or in the guided visit (see Paragraph 2.4.5).

*Phase 5*: Post-Treatment Assessment. At the conclusion of the session, participants returned to the conference room to complete post-intervention questionnaires. These corresponded to the pre-intervention instruments, with a final open-ended question asking participants to briefly describe their experience: “Come descriveresti la tua esperienza” (“How would you describe you experience”). Participants were told that their answers were part of the research and not a satisfaction survey. Phase 6: Debriefing. Upon completion of the questionnaires, participants were offered the opportunity to ask further questions to instructors and researchers and to receive information about future developments of the study and related initiatives.

### 2.4 Materials

The assessment battery comprised screening, trait, and state measures. The screening questionnaire collected demographic information (age, gender) and data concerning prior museum attendance and familiarity with the techniques employed in the project.

Trait measures were administered to assess baseline characteristics, including trait anxiety and perceived stress during the previous month.

State measures were employed to evaluate short-term changes associated with the intervention. The primary outcome variable was state anxiety, assessed immediately before and after the museum session. Additional psychological dimensions were measured using Visual Analogue Scales (see Section 2.4.3).

#### 2.4.1 Screening questionnaire

The screening instrument included items assessing inclusion and exclusion criteria, gender (woman, man, other, prefer not to say), age, level of interest in art or science museums (Likert scale from 1: not at all, to 10: very much), and annual museum attendance (never; 1–2 times; 3–5 times; >6 times).

#### 2.4.2 Trait measures

##### 2.4.2.1 TAI

The State and Trait Anxiety Inventory (STAI; Spielberger et al., 1970) is one of the most extensively validated instruments for the psychometric assessment of anxiety. The instrument comprises two separate 20-item scales measuring anxiety as Trait (TAI) and anxiety as State (SAI). Items are rated on a Likert scale ranging from 1 (not at all) to 4 (very much). In the recruitment phase, only the TAI was administered, whereas the SAI was employed during the pre- and post-assessment phases (see Section 2.4.3).

##### 2.4.2.2 PSS

The Perceived Stress Scale (PSS; Cohen et al., 1983) is a widely used measure of perceived stress. It assesses the degree to which individuals appraise life situations as unpredictable, uncontrollable, or overwhelming. Items refer to feelings and thoughts experienced over the previous month and are rated on a scale from 0 (never) to 4 (very often). Scores above 27 indicate high perceived stress, whereas scores below 14 indicate low perceived stress.

##### 2.4.2.3 BFI-10

BFI-10: the Italian short version (Guido et al., 2015) of the Big Five questionnaire (BFI-10) is a 10-item version (Rammstedt and John, 2007) of the more comprehensive BFI questionnaire (John et al., 1991) that contains 44 items. Participants are required to indicate their level of agreement on a scale ranging from 1 (“I do not agree at all”) to 5 (“I absolutely agree”). This questionnaire was developed to provide personality information in a very short time. Indeed, the BFI-10 allows the 5 personality traits to be assessed through only two items per dimension. Previous research has clearly shown that the BFI-10 possesses psychometric properties that are comparable in size and structure to those of the BFI in its standard version (Rammstedt et al., 2021). The BFI-10 assesses 5 traits: Openness, Agreeableness, Conscientiousness, Neuroticism, and Extraversion.

#### 2.4.3 State measures

##### 2.4.3.1 SAI

State anxiety was assessed before and after the museum activities using the SAI scale, following a pre–post protocol similar to that adopted by Binnie (2010). The SAI has demonstrated sensitivity to short-term changes in anxiety and robust psychometric properties (e.g., Field et al., 2013; Mackay and Neill, 2010; Vitasari et al., 2011). The scale comprises 20 items rated on a Likert scale from 1 (not at all) to 4 (very much), referring to the respondent’s current emotional state.

##### 2.4.3.2 Psychological assessment

Six visual-analogue scales (VAS) were presented to assess participants’ moods and other psychological dimensions before and after each museum session. The first question was about general mood: “How do you rate your mood right now?” with a scale from 1 (totally negative) to 10 (totally positive). The next 5 questions required assessing the intensity of certain states of mind experienced at the present moment, from 1 (absent state) to 10 (very present state), and specifically: stress; mental clarity; contentment; calmness; restlessness.

#### 2.4.4 VTS protocol

All VTS sessions were conducted by the same certified instructor following a standardised intervention protocol designed to ensure consistency across both museum settings. Participants attended the activity in groups of 8 to 16 individuals (one group per session; see Section 2.1); the spaces used were designed to accommodate groups up to 25 people. Activities were conducted while seated, with the surrounding space arranged to foster a focused, comfortable discussion environment. Each VTS session lasted approximately 1 h.

At each site, two museum objects were selected: one for an oral VTS discussion and one for a written VTS exercise. For the first object, the facilitator conducted an exclusively oral discussion. Participants were invited to observe the object (a painting, sculpture, or natural history exhibit), formulate hypotheses regarding its subject, and respond to the core VTS questions: “What’s going on in this picture?” and “What do you see that makes you say that?” Follow-up prompts included: “What more can you find?” and “Did you change your mind after listening to others?” Each participant was encouraged to articulate their interpretation and supporting reasoning, fostering shared meaning-making and cognitive flexibility.

For the second object, participants first completed a written VTS form (designed by the Italian VTS research group, Ferrara, 2016) that included the core questions and additional prompts on salient features and aspects they wished to explore further. Subsequently, an oral group discussion was facilitated, making sure that each participant presented and elaborated on their written reflections.

**Table:** 

Museum	Oral VTS discussion	Written VTS discussion
MAG	A painting	A sculpture
NHM	A sperm whale skeleton	A diorama

In all sessions conducted in both museums, the facilitator provided a factual description of the object only after the discussion had concluded, addressing questions that had emerged from the collective interpretative process. Further explanations were then provided by museum professionals not involved in the previous VTS session.

#### 2.4.5 Guided visit protocol

The comparison condition consisted of a standard guided visit, representing typical museum practice. The visit was led by an expert curator of the museum itself, who guided participants through a selection of the collection following the conventional expository format of a docent-led tour, providing factual and contextual information about the objects encountered. Unlike the VTS intervention, the standard visit did not employ structured open-ended questioning or facilitated group discussion; the museum expert delivered the content, while participants observed and could ask questions. To ensure comparability across conditions, the standard guided visit was matched to the VTS session in duration, globally lasting approximately 1 h and half, and was conducted with groups of comparable size (11–13 participants per session) at the same guides, one at MAG and one at NHM.

## 3 Statistical analysis

### 3.1 Overview and software

Statistical analyses were conducted using Python (version 3.12; SciPy, Virtanen et al., 2020) and SPSS 29.0 (IBM Corp., Armonk, NY). All tests were two-tailed, with *α* = 0.05. The *a priori* sample size was estimated for the primary outcome; anticipating possible dropouts, the target sample size was set at 35 participants per condition. Pre–post effect sizes are reported as d_av, that is, the mean change divided by the average of the pre- and post-intervention standard deviations (Lakens, 2013), with 95% bootstrap confidence intervals (3,000 resamples); d_z (the mean change divided by the standard deviation of the change scores) is also reported. For the six secondary VAS outcomes, *p*-values were adjusted with the Holm procedure (Holm, 1979); for the exploratory correlations between personality traits and change scores, the false discovery rate was controlled with the Benjamini–Hochberg procedure (Benjamini and Hochberg, 1995).

The sample size was estimated *a priori* in the study protocol (Lucchiari et al., 2024). Because research on museum-based activities and state anxiety was scarce and based on small samples, the calculation relied on data reported in previous studies using the State Anxiety Inventory in healthy adults (*M* = 38.5, SD = 10.9) and on the expectation of a 20% reduction in state anxiety (from 38.5 to 30.8) in the active condition, against no change in a control condition. Using Pocock’s formula for continuous outcomes (Pocock, 2013), *n = 2σ^2^/(μ2 − μ1)^2^ × f(α, β)*, with *f*(*α*, *β*) = 7.9 for *α* = 0.05 (two-tailed) and 80% power, 32 participants per group were required; this corresponds to a standardised between-group difference of *d* = 0.71. Anticipating attrition, the target was set at 35 participants per condition, and both conditions exceeded it (VTS: *n* = 85; guided visit: *n* = 50). Two points should be noted. First, the calculation was designed for a comparison between an active and a non-active condition; the present study compares two active museum activities and evaluates the pre–post change within each of them, so the analysis is powered for the within-condition change rather than for detecting small differences between the two activities. Second, the observed pre-intervention mean was lower than the normative value assumed in the protocol (*M* = 35.07), and the observed reduction corresponded to 15.8% of the baseline score. Furthermore, because a between-groups comparison requires a larger sample than a within-subjects pre–post comparison for the same standardised effect, the calculation was deliberately based on the more demanding between-groups scenario, so that the resulting sample size would also be adequate for the pre–post analyses.

### 3.2 Quantitative analyses

Demographic and trait variables (age, gender, museum attendance frequency, Big Five personality dimensions, Perceived Stress Scale, STAI-Trait, pre-intervention state anxiety, and perceived stress over the last month) were summarised using means and standard deviations for continuous variables and frequencies with percentages for categorical variables. Between-group comparisons on continuous variables (VTS vs. standard visit; MAG vs. NHM) were conducted using independent-samples *t*-tests with Welch’s correction for unequal variances. Categorical variables were compared using Pearson’s chi-square test. These analyses served to compare the two groups on baseline characteristics.

Changes in state anxiety (SAI) and the six VAS dimensions (mood, stress, clear-mindedness, contentment, calm, and restlessness) from pre- to post-intervention were first evaluated using paired-samples *t*-tests on complete pairs, for the total sample and separately for each condition and museum. Because participants attended the activities in groups and condition was assigned at the session level, observations may not be independent within sessions. We therefore estimated the intraclass correlation (ICC) of the change scores and re-estimated the main models as linear mixed models with a random intercept for session, estimated by restricted maximum likelihood in Python. Given the small number of sessions (11), tests of effects varying at the session level (condition, museum, and their interaction) used conservative degrees of freedom based on the number of sessions between-group comparisons of outcome changes. Post-intervention SAI scores were analysed with an ANCOVA including condition, museum, and their interaction as fixed factors and pre-intervention SAI as covariate, estimated both as a standard linear model and as a mixed model with a random intercept for session. We report adjusted means, the adjusted condition difference with its 95% confidence interval, and a test of the homogeneity of regression slopes (pre-intervention SAI × condition). For mood, the Time × Condition × Museum interaction was examined with a mixed ANOVA and then re-estimated on change scores with the multilevel approach described above. Differences between conditions in the remaining VAS change scores were examined descriptively.

Personality correlates of outcome changes. Pearson’s correlation coefficients were computed between the five Big Five trait scores (Extraversion, Agreeableness, Conscientiousness, Neuroticism, Openness) and the pre–post *Δ* for each outcome variable, for the total sample and separately by experimental condition. These analyses examined whether individual differences in personality were associated with responses to the museum intervention. Given the exploratory nature and the number of correlations computed (5 traits × 7 outcomes = 35 per group), *p-*values were adjusted with the Benjamini–Hochberg procedure within each group, and results should be treated as hypothesis-generating. All tests were two-tailed. All tests were two-tailed.

### 3.3 Content analysis of open-ended responses

Open-ended responses were brief (typically one to three sentences; median number of words = 35.68), which does not allow the in-depth interpretative work required by reflexive thematic analysis. We therefore used a qualitative content analysis with an inductive coding phase (Elo and Kyngäs, 2008; Hsieh and Shannon, 2005), aimed at describing rather than explaining participants’ experience. Two researchers (trained cognitive psychologist) independently read all responses, generated initial codes, and grouped them into categories, which were then compared and refined through discussion until consensus was reached. Ten categories were identified (see Section 4.2): fulfilment, immersion, learning, appreciation, uncertainty, relaxation, contemplation, socialisation, critical reflection, and guide.

To summarise each participant’s experience at a level suited to brief responses, each participant was assigned to the category that best characterised the overall response (dominant category), following explicit decision rules (e.g., responses combining the guide with emotionally valenced categories were assigned to guided emotions, and responses mentioning immersion to deep learning). Dominant categories were then grouped into three higher-order experiential routes reflecting our working hypothesis: cognitive (learning, deep learning, uncertainty, guided learning), emotional-aesthetic (appreciation, relaxation, contemplation, guided emotions), and social-relational (socialisation). The distribution of routes by condition is reported as proportions with 95% Wilson confidence intervals and was compared with a permutation chi-square test (10,000 permutations), which does not rely on large expected frequencies.

The two coders discussed all discrepancies until consensus. Given the brevity of the responses and the small size of several categories, associations between categories and outcome changes were not tested inferentially.

### 3.4 Missing data

All 135 participants completed the SAI at both time points. For the VAS, some participants left individual items unanswered, so that complete pre–post pairs ranged from 117 to 131 depending on the scale. Each analysis used all participants with complete data on the variables involved (available-case analysis), and the corresponding n is reported.

## 4 Results

### 4.1 Quantitative analyses

#### 4.1.1 Sample characteristics

The sample comprised 135 participants (VTS: *n* = 85; standard museum visit: *n* = 50), distributed across two museums: the Modern Art Gallery (MAG; *n* = 64) and the Natural History Museum (NHM; *n* = 71). The sample was predominantly female (82.2%), with a mean age of 44.86 years (*SD* = 18.03; range 19–89). Museum attendance was high: 43.9% of the 132 participants who reported this information visited museums more than six times per year; only four participants (3.0%) had never visited a museum.

Comparisons between the two experimental groups showed no significant differences in demographic variables: age (*t* = 1.55, *p* = 0.124), gender distribution (*χ*^2^ = 4.06, *p* = 0.132), and museum attendance frequency (*χ*^2^ = 3.80, *p* = 0.284). However, three baseline variables showed significant between-group imbalances (see Table 1). BFI Agreeableness was higher in the Visit group than in the VTS group (*M* = 5.91 vs. 4.80; *t* = −4.117, *p* < 0.001), as was pre-visit state anxiety (*M* = 36.83 vs. 34.05; *t* = −2.14, *p* = 0.034). Instead, Extraversion was higher in the VTS group (*M* = 6.13 vs. 4.92; *t* = 4.176, *p* < 0.001). These imbalances should be considered when interpreting between-group comparisons on outcome variables. In particular, the higher pre-visit state anxiety in the Visit group may have favoured a larger pre-to-post reduction through regression to the mean; this imbalance limits the interpretation of between-condition differences in state anxiety change. By contrast, trait anxiety and perceived stress did not differ significantly between the two groups.

**Table 1:** Sample characteristics by experimental condition.

Characteristic	Total (*n* = 135)	VTS (*n* = 85)	Visit (*n* = 50)	*t* or *χ*^2^	*p*
Age, *M* (SD)	44.9 (18.0)	46.7 (18.4)	41.7 (17.2)	1.55	0.124
Gender, *n* (%)					
Female	111 (82.2%)	72 (84.7%)	39 (78.0%)		
Male	21 (15.6%)	10 (11.8%)	11 (22.0%)		
Non-binary	3 (2.2%)	3 (3.5%)	0 (0.0%)	4.06	0.132ᵃ
Museum visits/year, *n* (%)					
Never	4 (3.05%)	1 (1.2%)	3 (6.0%)		
1–2 times	33 (25.4%)	24 (28.2%)	9 (18.0%)		
3–5 times	37 (28.5%)	23 (27.1%)	14 (28.0%)		
> 6 times	58 (42.9%)	37 (43.5%)	21 (42.0%)	3.80	0.284ᵃ
Trait measures, *M* (SD)					
STAI-Trait	44.8 (5.9)	44.2 (3.4)	45.8 (8.6)	−1.46	0.147
PSS	22.0 (6.4)	22.4 (6.6)	21.5 (5.9)	0.73	0.465
SAI (pre-visit)	35.1 (7.4)	34.1 (6.5)	36.8 (8.5)	−2.14	0.034*
BFI extraversion	5.7 (1.5)	6.13 (1.7)	4.92 (1.4)	4.18	<0.001***
BFI agreeableness	5.2 (1.5)	4.8 (1.4)	5.9 (1.4)	−4.12	<0.001***
BFI conscientiousness	5.4 (1.6)	5.3 (1.6)	5.5 (1.5)	−0.65	0.500
BFI neuroticism	5.8 (1.6)	5.8 (1.6)	5.8 (1.7)	0.96	0.924
BFI openness	4.2 (1.7)	4.2 (1.8)	4.3 (1.6)	−0.44	0.644

VTS, visual thinking strategies; PSS, Perceived Stress Scale; STAI-Trait, state–trait anxiety inventory, trait subscale; SAI, state anxiety subscale; BFI, big five inventory. *t*-tests are Welch-corrected independent-samples tests; ᵃchi-square test. *p < 0.05. ***p < 0.001. ^†^*p* < 0.10.

#### 4.1.2 Pre–post changes in state anxiety and mood

Across the total sample, state anxiety decreased from pre to post (M pre = 35.07, M post = 29.53; *Δ* = −5.54, 95% CI [−6.64, −4.44]; d_av = −0.81, 95% CI [−1.05, −0.60]; *t*(134) = −9.95, *p* < 0.001). All six VAS dimensions also changed in the expected direction, and all changes remained significant after Holm correction (all adjusted *p* ≤ 0.003): stress (*Δ* = −1.60; d_av = −0.75, 95% CI [−0.95, −0.58]), mood (*Δ* = +1.06; d_av = 0.72, [0.53, 0.93]), calm (*Δ* = +0.94; d_av = 0.57, [0.37, 0.79]), contentment (*Δ* = +0.84; d_av = 0.52, [0.34, 0.71]), restlessness (*Δ* = −0.82; d_av = −0.38, [−0.61, −0.18]), and clear-mindedness (*Δ* = +0.35; d_av = 0.21, [0.08, 0.35]). Dependence within sessions was negligible (ICC = 0.01 for ΔSAI and 0.04 for *Δ*mood). When session was modelled as a random effect, the reduction in state anxiety (*Δ* = −5.55, 95% CI [−6.85, −4.26], *t*(10) = −9.58, *p* < 0.001) and the improvement in mood (*Δ* = +1.07, 95% CI [0.74, 1.40], *t*(10) = 7.20, *p* < 0.001) remained robust.

Both conditions showed reductions in state anxiety (VTS: *Δ* = −4.76, 95% CI [−5.87, −3.66], d_av = −0.82; guided visit: *Δ* = −6.86, 95% CI [−9.18, −4.54], d_av = −0.84) and improvements in mood, stress, contentment, calm, and restlessness (Table 2).

**Table 2:** Pre–post changes in state anxiety (SAI) and mood (VAS) by group.

Scale	*n*	Pre *M* (SD)	Post *M* (SD)	*Δ* [95% CI]	*d*_av_ [95% CI]	*p*
Total sample (*N* = 135)						
State anxiety (SAI)	135	35.07 (7.36)	29.53 (6.25)	−5.54 [−6.64, −4.44]	−0.81 [−1.05, −0.60]	<0.001
Mood	131	7.02 (1.49)	8.08 (1.46)	+1.06 [0.82, 1.30]	+0.72 [0.53, 0.93]	<0.001
Stress	121	4.28 (2.31)	2.69 (1.93)	−1.60 [−1.94, −1.25]	−0.75 [−0.95, −0.58]	<0.001
Clear-mindedness	121	7.81 (1.80)	8.16 (1.56)	+0.35 [0.12, 0.58]	+0.21 [0.08, 0.35]	0.003
Contentment	121	7.23 (1.70)	8.07 (1.54)	+0.83 [0.56, 1.11]	+0.52 [0.34, 0.71]	<0.001
Calm	119	7.14 (1.79)	8.08 (1.53)	+0.94 [0.62, 1.26]	+0.57 [0.37, 0.79]	<0.001
Restlessness	117	3.38 (2.28)	2.56 (2.01)	−0.82 [−1.26, −0.38]	−0.38 [−0.61, −0.18]	<0.001
VTS (*n* = 85)						
State anxiety (SAI)	85	34.05 (6.45)	29.28 (5.11)	−4.76 [−5.87, −3.66]	−0.82 [−1.07, −0.63]	<0.001
Mood	81	6.95 (1.62)	8.14 (1.29)	+1.19 [0.93, 1.44]	+0.81 [0.65, 1.00]	<0.001
Stress	77	4.60 (2.42)	2.87 (2.00)	−1.73 [−2.15, −1.31]	−0.78 [−1.03, −0.59]	<0.001
Clear-mindedness	77	7.77 (1.90)	8.09 (1.54)	+0.32 [0.07, 0.58]	+0.19 [0.04, 0.34]	0.020
Contentment	77	7.08 (1.80)	7.88 (1.69)	+0.81 [0.43, 1.18]	+0.46 [0.24, 0.70]	<0.001
Calm	75	6.97 (1.95)	8.03 (1.57)	+1.05 [0.59, 1.52]	+0.60 [0.32, 0.91]	<0.001
Restlessness	75	3.59 (2.38)	2.75 (2.15)	−0.84 [−1.47, −0.21]	−0.37 [−0.69, −0.10]	0.020
Guided visit (*n* = 50)						
State anxiety (SAI)	50	36.82 (8.48)	29.96 (7.86)	−6.86 [−9.18, −4.54]	−0.84 [−1.27, −0.48]	<0.001
Mood	50	7.12 (1.26)	7.98 (1.70)	+0.86 [0.38, 1.34]	+0.58 [0.21, 1.07]	0.002
Stress	44	3.73 (2.03)	2.36 (1.78)	−1.36 [−1.98, −0.75]	−0.72 [−1.14, −0.40]	<0.001
Clear-mindedness	44	7.89 (1.62)	8.27 (1.60)	+0.39 [−0.08, 0.86]	+0.24 [−0.03, 0.57]	0.104
Contentment	44	7.50 (1.47)	8.39 (1.20)	+0.89 [0.49, 1.28]	+0.66 [0.40, 0.98]	<0.001
Calm	44	7.43 (1.45)	8.18 (1.47)	+0.75 [0.40, 1.10]	+0.51 [0.27, 0.83]	<0.001
Restlessness	42	3.00 (2.06)	2.21 (1.72)	−0.79 [−1.28, −0.29]	−0.42 [−0.75, −0.18]	0.006

SAI range: 20–80; VAS range: 1–10. *n* = participants with complete pre–post pairs. *Δ* = M post − M pre, with 95% confidence interval. *d*_av_ = *Δ* divided by the average of the pre- and post-intervention standard deviations (Lakens, 2013), with 95% percentile bootstrap confidence intervals (3,000 resamples). *p*-values are from paired-samples *t*-tests; for the six VAS outcomes they are adjusted within each group with the Holm procedure. Values by museum (MAG, NHM) are reported in Supplementary Table S1.

Clear-mindedness improved in the VTS group (*Δ* = +0.33, d_av = 0.19) but not in the guided-visit group (*Δ* = +0.39, 95% CI [−0.08, 0.86]). The larger raw reduction in state anxiety in the guided-visit group was accompanied by higher baseline anxiety. In the ANCOVA on post-intervention SAI, baseline SAI was a strong predictor (b = 0.46, 95% CI [0.33, 0.59]), and regression slopes did not differ between conditions (pre-intervention SAI × condition: *p* = 0.914). Adjusted post-intervention means were 29.61 for VTS and 29.23 for the guided visit; the adjusted difference (VTS − guided visit) was 0.38 points (95% CI [−2.67, 3.42], *t*(7) = 0.29, *p* = 0.780) in the multilevel model and 0.55 points (95% CI [−1.33, 2.42], *p* = 0.565) in the individual-level model. Neither museum nor the Condition × Museum interaction was significant in either model (all *p* ≥ 0.296). Thus, no statistically detectable difference between activities emerged after adjustment for baseline anxiety; given the width of the confidence interval, differences of up to about 3 SAI points in either direction cannot be excluded.

Both museums showed significant pre–post improvements on all measures except clear-mindedness at NHM (Table 2 and Figure 2), and no between-museum difference in change scores reached significance. For mood, descriptive cell means suggested an asymmetry: at MAG, the two activities showed similar improvements (*Δ* VTS = +1.13, *Δ* guided visit = +1.38), whereas at NHM the improvement was larger after VTS (*Δ* = +1.24) than after the guided visit (*Δ* = +0.38), a cell that also showed markedly higher variability (SD = 2.16, compared with 0.77–1.16 in the other cells). In the individual-level mixed ANOVA, the Time × Condition × Museum interaction was significant (*F*(1, 127) = 5.12, *p* = 0.025, *ηp*^2^ = 0.039); however, when session was taken into account, the corresponding Condition × Museum interaction on mood change was not significant (*b* = 1.11, 95% CI [−0.05, 2.27], *t*(7) = 2.26, *p* = 0.058), nor were the simple effects of condition within each museum (NHM: *p* = 0.116; MAG: *p* = 0.417). Given that each guided-visit cell included only two sessions, this pattern is reported as descriptive only.

**Figure 2:**
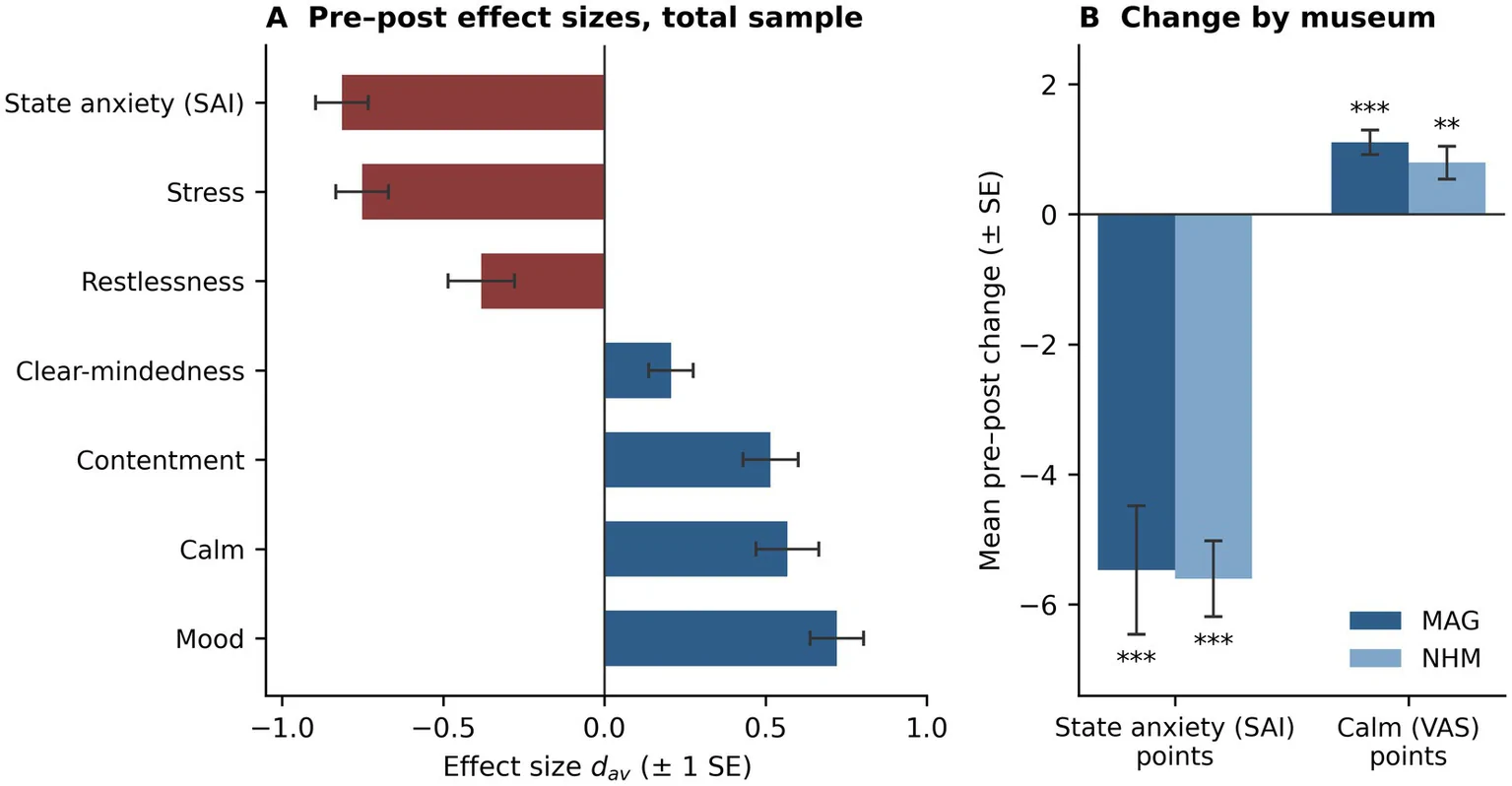
Pre–and post-changes in state anxiety and mood following the museum visit. Panel **(A)** shows standardised effect sizes (*d*_av) for all seven measures across the total sample (*N* = 117–135 complete pairs, depending on the scale; paired *t*-tests). Positive values indicate improvement (increase in positive states); negative values indicate reduction (decrease in negative states). Error bars represent ±1 SE of the mean delta. Panel **(B)** shows mean pre–post deltas (±SE) for state anxiety (SAI) and calm (VAS) separately for the two museum settings (MAG, Modern Art Gallery; NHM, Natural History Museum). All four bars reached significance (all *p* ≤ 0.003); the difference in the calm delta between museums did not reach statistical significance (*p* = 0.335). ***p < 0.001; **p < 0.01.

#### 4.1.3 Associations between personality traits and outcome changes

Pearson correlations between Big Five trait scores and pre–post *Δs* were computed for the total sample and separately by condition (Table 3). At the total-sample level, uncorrected *p-*values below 0.05 were observed only for the negative correlation between Agreeableness and *Δ*Calm (*r* = −0.20) and the positive correlation between Conscientiousness and *Δ*Restlessness (*r* = +0.20).

**Table 3:** Pearson correlations between big five traits and pre–post outcome deltas (total sample).

Personal trait	ΔSAI	*Δ*Mood	*Δ*Stress	*Δ*Clear	*Δ*Content	*Δ*Calm	*Δ*Restless
Extraversion	−0.04	−0.04	−0.06	+0.10	−0.11	−0.12	+0.01
Agreeableness	−0.03	−0.06	−0.06	+0.08	−0.15^†^	−0.20*	−0.05
Conscientiousness	−0.04	−0.10	+0.06	−0.14	−0.12	−0.15	+0.20^*^
Neuroticism	+0.06	+0.13	−0.16^†^	+0.05	−0.08	−0.01	+0.02
Openness	−0.14	+0.03	−0.12	+0.10	+0.01	−0.09	+0.03

*n* = 127–135 depending on scale. *Δ*SAI = delta in state anxiety (post − pre). *Δ*Clear = *Δ* clear-mindedness. *p < 0.05. ^†^*p* < 0.10. Uncorrected *p*-values; no correlation survived the Benjamini–Hochberg correction. Results are exploratory.

Within the VTS group, uncorrected *p-*values of 0.05 or lower were observed for the negative correlations of Agreeableness (*r* = −0.28) and Extraversion (*r* = −0.23) with *Δ*Calm, and for the positive correlation of Conscientiousness with *Δ*Restlessness (*r* = +0.27): more agreeable and more extraverted participants showed smaller gains in calm, and more conscientious participants showed smaller reductions in restlessness. No correlation reached *p* < 0.05 in the guided-visit group.

Overall, Big Five traits accounted for a modest proportion of variance in outcome changes, and none of the correlations survived the Benjamini–Hochberg correction (smallest adjusted *p* = 0.38 in the VTS group and 0.61 in the total sample). These associations are therefore reported only as descriptive patterns that may inform future hypotheses.

### 4.2 Qualitative analyses of participants’ experience

Participants’ open-ended responses were analysed as described in Section 3.3. The 10 categories identified are defined below.

- *Fulfilment*. Describes a sense of completeness and personal reward that participants experienced at the end of the museum visit. Unlike mere aesthetic appreciation, which refers to the pleasure derived from the artwork itself, fulfilment implies a positive evaluation of the visit as a whole: participants felt the experience had given them something meaningful, whether on a cognitive, emotional, or personal level. In its more intense form, this theme conveys a transformative quality, as though the visit had responded to an inner need or brought something to a conclusion. In its less intense form, closer to satisfaction, it manifests as a sense of closure and positive fulfilment of expectations. This theme was reported exclusively in the Visit group, suggesting that the narrative, museum expert-led format of the standard guided visit, which presents the collection as a coherent whole may facilitate this kind of integrative, conclusive experience.
- *Immersion*. In the VTS group, this theme has a strong cognitive nuance and is described by participants as a boost to understanding. In fact, perception seems to be enriched through explanation. Also, meaning gradually emerges through observation, encompassing both the details and the most coherent way of integrating them into a unified whole. In the VISIT group, identification, empathy, creativity, and passion were frequently mentioned as ways to engage deeply with the artworks. The experience was described as immersive, as though participants were entering the artworks themselves and emerging enriched.
- *Learning*. In the VTS group, this theme was primarily articulated in relation to listening to the professionals’ explanations, which participants described as highly engaging and informative. In contrast, in the VISIT group, listening itself was not explicitly mentioned; instead, greater emphasis was placed on the learning process, which culminated in an enriching experience.
- *Appreciation*. It refers to an aesthetic response to the artworks encountered during the visit, characterised by a sense of pleasure, wonder, or recognition of beauty. It represents one of the most fundamental and pervasive dimensions of the museum experience, appearing in both groups and across a wide range of combinations with other themes.
  In the VTS group, appreciation tends to emerge as a mediated and elaborated response: the dialogical structure of the visit encourages participants to dwell on the artwork, articulate observations, and construct a shared aesthetic judgement. Appreciation here is often the outcome of a reflective or social process; it frequently co-occurs with immersion, socialisation, and reflection, rather than a spontaneous reaction. Participants seem to arrive at appreciation through engagement, as though the act of looking carefully and discussing what is seen gradually reveals the work’s value.
  In the Visit group, appreciation takes a more immediate and personal form: participants report direct aesthetic pleasure, often linked to the visual beauty of the works, their evocative power, or their resonance with personal experience. When appreciation appears as the sole theme (which is more common in this group), it signals a self-contained, unmediated encounter with the artwork that requires no further elaboration.
- *Uncertainty*. This theme was reported predominantly within the VTS group. Here, likely because the intervention differed from a traditional museum visit, participants described a gradual process of understanding, almost like a roller coaster experience in which learning was intertwined with moments of anticipation and uncertainty.
- *Relaxation*. The theme of relaxation was reported by participants in both museums, almost exclusively in the Visit condition, where it was described as a pleasant sense of physical and mental relief in both museums.
- *Contemplation*. it refers to a quiet, inward-looking mode of engagement with the artwork, in which the experience itself becomes an occasion for personal resonance and inner elaboration. Rather than analysing or evaluating, the participant pauses, allows the work to settle, and finds that it activates thoughts, memories, or feelings that extend beyond the artwork itself. The process is largely spontaneous and non-linear: meaning does not need to be reached or articulated; it is enough that something has been stirred.
- *Socialisation*. The theme of social interaction was articulated in a particularly nuanced manner, especially within the VTS group. Participants highlighted both the playful dimension of interacting with others and the sense of sharing that emerged, almost as though the multiplicity of narratives had fostered a feeling of horizontality, closeness, and equality. They also referred to a sense of group unity and emotional contagion that shaped the overall experience, as well as to how the presence of others amplified it and drew attention to aspects that might otherwise have gone unnoticed. Although the theme was also mentioned in the VISIT group, it was discussed less frequently and more superficially.
- *Critical reflection*. it describes an analytically distanced mode of engagement in which the participant steps back from the immediate experience and evaluates the artwork, or the visit itself, against a framework of prior knowledge, expectations, or aesthetic criteria. Unlike contemplation, which is immersive and non-judgmental, critical reflection involves an explicit cognitive effort to assess, question, or reinterpret: participants may challenge their initial reading of the work, consider alternative perspectives, or notice a gap between what they expected and what they encountered.
  In the VTS group, critical reflection appears to be fostered, perhaps paradoxically, by the dialogical structure of the visit: the facilitator’s open-ended questions encourage participants to justify their observations and consider other viewpoints, which naturally generates a meta-cognitive stance toward the work.
  In the Visit group, critical reflection emerges more sporadically and seems to reflect a pre-existing intellectual disposition in the visitor rather than a situationally induced response. When present, it tends to appear as a solo theme; the visit as a whole is experienced primarily through this analytical lens and is more likely to involve comparison with personal knowledge or prior exposure to the artwork.
  The key distinction from contemplation is directional: contemplation is a movement inward, allowing the artwork to resonate with the self; critical reflection is a movement outward and upward, positioning the self as an evaluating subject in relation to the work.
- *Guide*. In the VISIT group, participants highlighted guide’s dialogical skills and expertise but also relational qualities such as empathy, passion, and kindness. This theme was not present in the VTS group.

#### 4.2.1 Experiential routes by condition

Table 4 reports the distribution of the three experiential routes, based on each participant’s dominant category. After VTS, most participants described their experience in cognitive terms (58.8%, 95% CI [48.2, 68.7]), followed by social-relational (22.4%, [14.8, 32.3]) and emotional-aesthetic descriptions (18.8%, [11.9, 28.4]). After the guided visit, emotional-aesthetic descriptions prevailed (58.0%, [44.2, 70.6]), followed by cognitive ones (42.0%, [29.4, 55.8]), whereas no participant gave a predominantly social-relational description (0%, [0.0, 7.1]). The distribution differed between conditions (permutation *χ*^2^(2) = 27.37, *p* < 0.001; Cramér’s V = 0.45), and the same overall pattern was observed in both museums (Table 4).

**Table 4:** Distribution of experiential routes by condition and museum.

Condition	Museum	Route	*n*	Total	%	95% CI low	95% CI high
VTS	Both	Cognitive	50	85	58.8	48.2	68.7
VTS	Both	Emotional-aesthetic	16	85	18.8	11.9	28.4
VTS	Both	Social-relational	19	85	22.4	14.8	32.3
VTS	MAG	Cognitive	23	40	57.5	42.2	71.5
VTS	MAG	Emotional-aesthetic	5	40	12.5	5.5	26.1
VTS	MAG	Social-relational	12	40	30	18.1	45.4
VTS	NHM	Cognitive	27	45	60	45.5	73
VTS	NHM	Emotional-aesthetic	11	45	24.4	14.2	38.7
VTS	NHM	Social-relational	7	45	15.6	7.7	28.8
Guided visit	Both	Cognitive	21	50	42	29.4	55.8
Guided visit	Both	Emotional-aesthetic	29	50	58	44.2	70.6
Guided visit	Both	Social-relational	0	50	0	0	7.1
Guided visit	MAG	Cognitive	9	24	37.5	21.2	57.3
Guided visit	MAG	Emotional-aesthetic	15	24	62.5	42.7	78.8
Guided visit	MAG	Social-relational	0	24	0	0	13.8
Guided visit	NHM	Cognitive	12	26	46.2	28.8	64.5
Guided visit	NHM	Emotional-aesthetic	14	26	53.8	35.5	71.2
Guided visit	NHM	Social-relational	0	26	0	0	12.9

Permutation chi-square (condition × route): *χ*^2^(2) = 27.37, *p* < 0.001 (10,000 permutations); Cramér’s *V* = 0.45. Routes based on the dominant category (MainTheme).

At the level of individual categories, deep learning was the most frequent dominant category after VTS (33 of 85 participants), followed by socialisation (19) and uncertainty (11); after the guided visit, the most frequent were learning (11), relaxation (11), and guided emotions (9). Guided learning and guided emotions, which by definition involve the guide, occurred only in the guided-visit condition.

## 5 Discussion

The present study examined whether two museum activities designed for educational purposes, VTS and a standard guided visit, were associated with short-term changes in state anxiety and mood in two different museum settings. Both activities were followed by significant reductions in state anxiety, the primary outcome, and by improvements in all VAS measures in the total sample. These findings are consistent with previous studies reporting reductions in anxiety and stress after museum visits and art viewing (Binnie, 2010; Grossi et al., 2019; Banzi et al., 2026) and with broader reviews on the health benefits of arts engagement (Fancourt and Finn, 2019). However, because the design did not include a non-museum control condition, the observed changes cannot be causally attributed to the museum activities or to the museum environment. Alternative explanations, including the effect of taking part in a structured one-hour group activity, participants’ expectations, demand characteristics, and regression to the mean, cannot be ruled out.

Regarding the comparison between activities, the guided visit showed a numerically larger reduction in state anxiety than VTS, but this difference largely reflected the higher baseline anxiety of the guided-visit group: after adjustment for baseline anxiety, and taking sessions into account, the adjusted difference between activities was 0.38 SAI points (95% CI [−2.67, 3.42]). The present data therefore provide no statistically detectable evidence of a difference between the two activities; they do not show that the activities are equivalent, and differences of a few SAI points in either direction remain compatible with the data.

The content analysis adds a qualitative dimension to these findings. Although the two activities were followed by comparable changes in state anxiety, participants described them in different terms: after VTS, descriptions were mainly cognitive and, for about one participant in five, social-relational, whereas after the guided visit they were mainly emotional-aesthetic, and social-relational descriptions were absent. This pattern is consistent with our working hypothesis that different museum activities may lead to similar short-term outcomes through different experiential routes. VTS, by asking participants to observe, justify their interpretations, and listen to others, may engage visitors mainly through reasoning and shared meaning-making, whereas the expert-led visit may engage them mainly through aesthetic appreciation, relaxation, and emotional resonance with the guide’s narrative. Two cautions apply. First, the way participants describe an experience partly reflects what the activity asks them to do: VTS explicitly trains participants to verbalise their reasoning, which may favour cognitive descriptions regardless of the underlying process. Second, the responses were brief and the categories were not linked to outcome changes, so the routes are descriptive and hypothesis-generating. For museum professionals, however, the pattern may be useful: it suggests that activities with different formats can offer visitors different ways into a similar experience, and that the choice of format could be matched to visitors’ preferences or to the goals of a programme.

A further interpretative consideration concerns the between-group differences in Extraversion and Agreeableness. The VTS group scored significantly higher on Extraversion, whereas the Visit group scored higher on Agreeableness. These differences were not anticipated at the design stage and likely reflect the self-selection dynamics of opportunistic recruitment rather than any systematic confound; nonetheless, they may have shaped the experiential quality of each condition in theoretically meaningful ways. Individuals higher in Agreeableness tend to orient positively towards social guidance, to be receptive to expert authority, and to derive satisfaction from harmonious, externally structured interactions,. qualities that are consistent with the emotional-aesthetic and guide-related categories (guided emotions, relaxation, fulfilment) that characterised the Visit condition. Conversely, higher Extraversion in the VTS group may have facilitated engagement with the dialogical, group-based format. Yet, as the within-VTS correlations tentatively suggest (although they did not survive correction for multiple comparisons), it may simultaneously have attenuated gains in calm and contentment, insofar as socially oriented individuals bring a degree of interpersonal activation to structured group contexts that is not always conducive to affective quieting. Taken together, this pattern suggests that personality may co-determine not only the magnitude but also the qualitative texture of the museum experience. In fact, the themes that emerged most frequently in each condition (deep learning and socialisation in VTS; guided emotions and relaxation in Visit) are consistent with the dispositional profiles of the respective groups. Both conditions were nonetheless followed by improvements in anxiety and mood despite these differences in trait composition. Nevertheless, this pattern suggests that future studies should incorporate random assignment with stratification on key personality dimensions, so that the independent contributions of intervention format and visitor disposition can be properly disentangled.

The fact that neither activity was designed to reduce anxiety makes these findings of practical interest: both VTS and the standard guided visit are educational and interpretative formats, yet each was followed by a reduction in state anxiety, the single confirmatory outcome for which the study was powered. The study was not designed to test the superiority of VTS over the standard visit. One possible interpretation of the similar pattern observed in the two conditions is that features shared by both activities, such as slowing down, focused attention on objects, a group setting, the museum atmosphere, and the factual information provided by museum professionals (throughout the guided visit and at the end of the VTS session), contribute to the observed changes. This interpretation is consistent with frameworks describing museums as settings that can support wellbeing (Chatterjee and Camic, 2015; Cotter and Pawelski, 2021), but it remains a hypothesis to be tested in studies comparing museum activities with matched activities carried out in non-museum settings.

The descriptive asymmetry observed for mood, a smaller improvement after the guided visit at NHM, deserves mention but not interpretation: it did not survive a multilevel analysis accounting for sessions, and each guided-visit cell included only two sessions. If replicated in studies with more sessions, it would suggest that the fit between activity format and type of collection may matter, for instance because a dialogical format may make less familiar objects, such as a skeleton or a diorama, more engaging than an expository tour.

The associations between Big Five personality traits and outcome changes. Were modest, primarily confined to the VTS condition, and did not survive correction for multiple comparisons; they may nonetheless suggest a line of inquiry. Within the VTS group, the negative correlations of extraversion and agreeableness with gains in calm, and the positive correlation between conscientiousness and change in restlessness, may suggest that individuals with these dispositions engage with the VTS dialogical structure in ways that are cognitively activating rather than relaxing, possibly because the structured format creates implicit performance expectations. If replicated, this could support tailoring structured museum interventions to individual difference profiles or offering participants within VTS sessions explicit permission to engage at their own pace.

Several limitations qualify the present findings. First and most importantly, the study did not include a neutral, non-museum control condition, since both activities took place in a museum. Without such a control, the observed pre–post changes cannot be causally attributed to the museum context or to the specific activities, and may partly reflect non-specific factors such as group participation, expectations, demand characteristics, and regression to the mean. Second, participants were not randomly assigned to conditions but were allocated according to the session date they selected, without knowing which activity would be delivered; the two groups differed in size, baseline state anxiety, extraversion, and agreeableness, and adjustment for baseline anxiety cannot remove unmeasured confounding. Third, condition was assigned at the session level and the number of sessions was small (seven VTS and four guided-visit sessions). Although dependence within sessions was negligible and the main results were unchanged in multilevel models, comparisons between conditions rest on few sessions and should be interpreted with caution. Fourth, each activity was delivered by a single professional (the same VTS facilitator and the same museum guide throughout), so that activity format and facilitator are confounded. Fifth, a non-significant difference between conditions does not establish that the two activities are equivalent, and the confidence interval of the adjusted difference was compatible with differences of up to about 3 SAI points in either direction. Sixth, all outcomes were self-reported and assessed immediately after the session; the absence of a follow-up assessment prevents any conclusions about the durability of the observed changes. Seventh, the open-ended responses were brief, so the content analysis is descriptive and cannot establish the processes underlying the observed changes. Furthermore, the sample was predominantly female, attended museums frequently, and was self-selected, which restricts the generalisability of the findings to broader or more diverse populations. Finally, within the ASBA protocol, VTS and the standard guided visit were included as activities not designed for anxiety reduction, to be compared with activities of established anxiolytic value (e.g., mindfulness, art therapy) delivered in the same museums. The present study reports the former; the comparison between the two activities was therefore not the primary aim of the design, and the study was not powered to detect small differences between them.

Future research should address these limitations through randomised controlled designs that include non-museum comparison conditions and a larger number of sessions per condition, longitudinal follow-ups, larger and more heterogeneous samples, several facilitators per format, and physiological measures alongside self-report outcomes. The experiential routes suggested by the content analysis could be tested directly, for instance with brief validated measures of cognitive, emotional, and social engagement administered after the activity and related to outcome changes. More broadly, what participants make of the visit, not merely what they are offered, may be an important determinant of affective change and deserves to be studied as such.

## 6 Conclusion

In sum, both a structured VTS session and a standard guided visit were followed by short-term reductions in state anxiety and improvements in mood in two different museums, with no statistically detectable difference between the two activities. Participants, however, described the two experiences in different terms, predominantly cognitive after VTS and emotional-aesthetic after the guided visit. These preliminary findings do not establish that museum activities cause these changes, but they justify controlled studies testing whether, and through which routes, educational museum activities can contribute to visitors’ wellbeing.

## Data Availability

The raw data supporting the conclusions of this article will be made available by the authors, without undue reservation.

## References

[ref1] Banzi (2023). The Brain-Friendly Museum: Using Psychology and Neuroscience to Improve the Visitor Experience. New York-London: Routledge.

[ref2] Banzi A. Sacco P. L. Vanutelli M. E. Lucchiari C. (2026). The museum as a mindful space: reducing visitors’ stress and anxiety levels through the ASBA protocol. Behav. Sci. 16:116. doi: 10.3390/bs16010116, 41595058 PMC12838231

[ref3] Benjamini Y. Hochberg Y. (1995). Controlling the false discovery rate: a practical and powerful approach to multiple testing. J. R. Stat. Soc. B Stat. Methodol. 57, 289–300. doi: 10.1111/j.2517-6161.1995.tb02031.x

[ref4] Binnie J. (2010). Does viewing art in the museum reduce anxiety and improve wellbeing? Museums Soc. Issues 5, 191–201. doi: 10.1179/msi.2010.5.2.191

[ref5] Bolwerk A. Mack-Andrick J. Lang F. R. Dörfler A. Maihöfner C. (2014). How art changes your brain: differential effects of visual art production and cognitive art evaluation on functional brain connectivity. PLoS One 9:e101035. doi: 10.1371/journal.pone.0101035 PMC4077746 24983951

[ref6] Cardillo E. R. Chatterjee A. (2025). Benefits of nature imagery and visual art in healthcare contexts: a view from empirical aesthetics. Buildings 15:1027. doi: 10.3390/buildings15071027

[ref7] Cerqueira A. R. Alves A. S. Monteiro-Soares M. Hailey D. Loureiro D. Baptista S. (2023). Visual thinking strategies in medical education: a systematic review. BMC Med. Educ. 23:536. doi: 10.1186/s12909-023-04470-3, 37501147 PMC10375761

[ref8] Chatterjee H. J. Camic P. M. (2015). The health and wellbeing potential of museums and art galleries. Arts Health 7, 183–186. doi: 10.1080/17533015.2015.1065594

[ref9] Cohen S. Kamarck T. Mermelstein R. (1983). A global measure of perceived stress. J. Health Soc. Behav. 24, 385–396. doi: 10.2307/2136404, 6668417

[ref10] Cotter K. N. Alpys A. Rosenberg J. Rodriguez-Boerwinkle R. M. Pawelski J. O. (2024). The well-being benefits of virtual art galleries: examining the roles of emotion, immersion, and individual differences. Int. J. Wellbeing 14, 1–22. doi: 10.5502/ijw.v14i2.3603

[ref11] Cotter K. N. Pawelski J. O. (2021). Art museums as institutions for human flourishing. J. Posit. Psychol. 17, 288–302. doi: 10.1080/17439760.2021.2016911

[ref12] Desmarais S. Bedford L. Chatterjee H. J. (2018). Museums as Spaces for Wellbeing: A Second Report from the National Alliance for Museums, Health and Wellbeing. London: National Alliance for Museums, Health and Wellbeing.

[ref13] Dewey J. (1934). Art as Experience. New York: Perigee.

[ref14] Dow R. Warran K. Letrondo P. Fancourt D. (2023). The arts in public health policy: progress and opportunities. Lancet Public Health 8, e155–e160. doi: 10.1016/S2468-2667(22)00313-9, 36709054

[ref15] Elo S. Kyngäs H. (2008). The qualitative content analysis process. J. Adv. Nurs. 62, 107–115. doi: 10.1111/j.1365-2648.2007.04569.x, 18352969

[ref16] Fancourt D. Finn S. (2019). What is the evidence on the role of the arts in improving health and well-being? A scoping review. WHO Regional Office for Europe. Health Evidence Network (HEN) synthesis report 67. who.int. 32091683

[ref17] Ferrara V. (2016). Arte e Apprendimento - Strategie di pensiero visuale. Roma: Digilab Sapienza.

[ref18] Ferrara V. (2020). L’arte come strumento per la formazione in area medica e sanitaria. Roma: Aracne Editore.

[ref19] Ferrara V. Perrone M. E. Mina C. Ceccarelli F. Olivieri G. Fattapposta F. . (2022b). Science and visual arts: binomial or dichotomy? A pilot study in systemic lupus erythematosus patients. Lupus 31, 1744–1749. doi: 10.1177/09612033221131179 36189670

[ref20] Ferrara V. Shaholli D. Iovino A. Cavallino S. Colizzi M. A. Della Rocca C. . (2022a). Visual thinking strategies as a tool for reducing burnout and improving skills in healthcare workers: results of a randomized controlled study. J. Clin. Med. 11:7501. doi: 10.3390/jcm11247501, 36556117 PMC9784727

[ref21] Field T. Diego M. Delgado J. Medina L. (2013). Peer support and interpersonal psychotherapy groups experienced decreased prenatal depression, anxiety and cortisol. Early Hum. Dev. 89, 621–624. doi: 10.1016/j.earlhumdev.2013.04.006 PMC3742661 23727060

[ref22] Fudickar A. Konetzka D. Nielsen S. M. L. Hathorn K. (2022). Evidence-based art in the hospital. Wien. Med. Wochenschr. 172, 234–241. doi: 10.1007/s10354-021-00861-7 PMC8326640 34338902

[ref23] Golden T. L. Sonke J. Rodriguez A. K. (2024). An evidence-based framework for the use of arts and culture in public health. Health Promot. Pract. 26, 454–462. doi: 10.1177/15248399241228831, 38374701

[ref24] Grossi E. Tavano Blessi G. Sacco P. L. (2019). Magic moments: determinants of stress relief and subjective wellbeing from visiting a cultural heritage site. Cult. Med. Psychiatry 43, 4–24. doi: 10.1007/s11013-018-9593-8. 29998384, 29998384

[ref25] Guido G. Peluso A. M. Capestro M. Miglietta M. (2015). An Italian version of the 10-item big five inventory: an application to hedonic and utilitarian shopping values. Pers. Individ. Differ. 76, 135–140. doi: 10.1016/j.paid.2014.11.053

[ref27] Holm S. (1979). A simple sequentially rejective multiple test procedure. Scand. J. Stat. 6, 65–70.

[ref28] Housen A. C. (2002). Aesthetic thought, critical thinking and transfer. Arts Learn. Res. J. 18, 99–132.

[ref29] Hsieh H.-F. Shannon S. E. (2005). Three approaches to qualitative content analysis. Qual. Health Res. 15, 1277–1288. doi: 10.1177/1049732305276687, 16204405

[ref30] Iosa M. Salera C. Capua C. De Angelis D. Coiro P. Venturiero V. . (2022). Inside the Michelangelo effect: the role of art and aesthetic attractiveness on perceived fatigue and hand kinematics in virtual painting. Psych. J. 11, 695–707. doi: 10.1002/pchj.606 PMC9827837 36168965

[ref31] Jensen A. Bonde L. O. (2018). The use of arts interventions for mental health and wellbeing in health settings. Perspect. Public Health 138, 209–214. doi: 10.1177/1757913918772602, 29708025

[ref32] John O. P. Donahue E. M. Kentle R. L. (1991). The Big Five Inventory—Versions 4a and 54. Berkeley, CA: University of California, Berkeley, Institute of Personality and Social Research.

[ref33] Klugman C. M. Peel J. Beckmann-Mendez D. (2011). Art rounds: teaching interprofessional students visual thinking strategies at one school. Acad. Med. 86, 1266–1271. doi: 10.1097/ACM.0b013e31822c1427, 21869658

[ref34] Lakens D. (2013). Calculating and reporting effect sizes to facilitate cumulative science: a practical primer for t-tests and ANOVAs. Front. Psychol. 4:863. doi: 10.3389/fpsyg.2013.00863, 24324449 PMC3840331

[ref35] Lucchiari C. Vanutelli M. E. Ferrara V. Folgieri R. Banzi A. (2024). Promoting wellbeing in the museum: the ASBA project research protocol. Int. J. Health Wellness Soc. 14, 73–88. doi: 10.18848/2156-8960/cgp/v14i04/73-88

[ref36] Mackay G. J. Neill J. T. (2010). The effect of “green exercise” on state anxiety and the role of exercise duration, intensity, and greenness: a quasi-experimental study. Psychol. Sport Exerc. 11, 238–245. doi: 10.1016/j.psychsport.2010.01.002

[ref37] Montessori M. (1909). Il metodo della pedagogia scientifica applicato all'educazione infantile nelle case dei bambini. Città di Castello: Scipione Lapi.

[ref38] Piaget J. (2000). Lo sviluppo mentale del bambino e altri studi di psicologia. Torino: Einaudi.

[ref39] Pocock S. J. (2013). Clinical Trials: A Practical Approach. New York: John Wiley & Sons.

[ref40] Poirier T. I. Newman K. Ronald K. (2020). An exploratory study using visual thinking strategies to improve undergraduate students’ observational skills. Am. J. Pharm. Educ. 84:7600. doi: 10.5688/ajpe7600, 32431308 PMC7223938

[ref41] Rammstedt B. John O. P. (2007). Measuring personality in one minute or less: a 10-item short version of the big five inventory in English and German. J. Res. Pers. 41, 203–212. doi: 10.1016/j.jrp.2006.02.001

[ref42] Rammstedt B. Lechner C. M. Danner D. (2021). Short forms do not fall short: a comparison of three (extra-) short forms of the big five. Eur. J. Psychol. Assess. 37:23. doi: 10.1027/1015-5759/a000574

[ref43] Spielberger C. D. Gorsuch R. L. Lushene R. E. (1970). Manual for the State-Trait Anxiety Inventory. Palo Alto, CA: Consulting Psychologists Press.

[ref44] van Leeuwen J. E. P. Crutch S. J. Warren J. D. (2023). Thinking eyes: visual thinking strategies and the social brain. Front. Psychol. 14:1222608. doi: 10.3389/fpsyg.2023.1222608, 37829065 PMC10565500

[ref45] Vanutelli M. E. Banzi A. Cicirello M. Folgieri R. Lucchiari C. (2025b). Predicting state anxiety level change using EEG parameters: a pilot study in two museum settings. Brain Sci. 15:855. doi: 10.3390/brainsci15080855, 40867186 PMC12384159

[ref46] Vanutelli M. E. Banzi A. Lucchiari C. (2025a). Employees wellbeing and yoga. The case of the Museum of Asian art staff. Wellbeing Futures Innov. Policy Res. 1, 72–82. doi: 10.65638/2978-882x.2025.01.06

[ref47] Virtanen P. Gommers R. Oliphant T. E. Haberland M. Reddy T. Cournapeau D. . (2020). SciPy 1.0: fundamental algorithms for scientific computing in Python. Nat. Methods 17, 261–272. doi: 10.1038/s41592-019-0686-2, 32015543 PMC7056644

[ref48] Vitasari P. Wahab M. N. A. Herawan T. Othman A. Sinnadurai S. K. (2011). Re-test of state trait anxiety inventory (STAI) among engineering students in Malaysia: reliability and validity tests. Procedia. Soc. Behav. Sci. 15, 3843–3848. doi: 10.1016/j.sbspro.2011.04.383

[ref49] Vygotskij L. S. (1934). Pensiero e Linguaggio - ricerche psicologiche, traduzione di Luciano (1990) Mecacci. Roma: Laterza.

[ref50] WHO. (1999). Partners in Life Skills Education: Conclusions from a United Nations Inter-Agency Meeting. Available online at: https://www.orientamentoirreer.it/sites/default/files/materiali/1999%20OMS%20lifeskills%20edizione%201999.pdf (accessed July 1, 2026)

[ref51] Yenawine P. (2013). Visual Thinking Strategies: Using Art to Deepen Thinking across school Disciplines. Cambridge, MA: Harvard Education Press.

[ref52] Yifei S. Nasri N. (2025). Art education and social emotional learning: practices to enhance students' self-awareness and sense of social responsibility. Int. J. Acad. Res. Progressive Educ. Dev. 14, 2315–2330. doi: 10.6007/IJARPED/v14-i4/27222

