# Supplementary material for: Museum-based activities and psychological wellbeing: a pre–post study of visual thinking strategies and guided visits across two cultural settings
Source: Front Psychol. 2026 Oct 19;17:1904352. doi: 10.3389/fpsyg.2026.1904352 (PMC13724324; doi:10.3389/fpsyg.2026.1904352)
Supplement: Supplementary file 1 [file Table_1.DOCX]

Supplementary Table S1

*Pre–Post Changes in State Anxiety (SAI) and Mood (VAS) by Group and by Museum: Full Statistics*

| Scale | *n* | Pre M (SD) | Post M (SD) | Δ | Δ 95% CI | SD of Δ | *d_av_* | *d_av_* 95% CI | *d_z_* | *t* | *df* | *p* | *p Holm* |
| --- | --- | --- | --- | --- | --- | --- | --- | --- | --- | --- | --- | --- | --- |
| *Total sample (N = 135)* |  |  |  |  |  |  |  |  |  |  |  |  |  |
| State anxiety (SAI) | 135 | 35.07 (7.36) | 29.53 (6.25) | −5.54 | [−6.64, −4.44] | 6.47 | −0.81 | [−1.05, −0.60] | −0.86 | −9.95 | 134 | < .001 | — |
| Mood | 131 | 7.02 (1.49) | 8.08 (1.46) | +1.06 | [0.82, 1.30] | 1.39 | +0.72 | [0.53, 0.93] | +0.76 | 8.73 | 130 | < .001 | < .001 |
| Stress | 121 | 4.28 (2.31) | 2.69 (1.93) | −1.60 | [−1.94, −1.25] | 1.91 | −0.75 | [−0.95, −0.58] | −0.83 | −9.17 | 120 | < .001 | < .001 |
| Clear-mindedness | 121 | 7.81 (1.80) | 8.16 (1.56) | +0.35 | [0.12, 0.58] | 1.28 | +0.21 | [0.08, 0.35] | +0.27 | 2.99 | 120 | .003 | .003 |
| Contentment | 121 | 7.23 (1.70) | 8.07 (1.54) | +0.83 | [0.56, 1.11] | 1.53 | +0.52 | [0.34, 0.71] | +0.55 | 6.00 | 120 | < .001 | < .001 |
| Calm | 119 | 7.14 (1.79) | 8.08 (1.53) | +0.94 | [0.62, 1.26] | 1.76 | +0.57 | [0.37, 0.79] | +0.53 | 5.83 | 118 | < .001 | < .001 |
| Restlessness | 117 | 3.38 (2.28) | 2.56 (2.01) | −0.82 | [−1.26, −0.38] | 2.39 | −0.38 | [−0.61, −0.18] | −0.34 | −3.71 | 116 | < .001 | < .001 |
| *VTS (n = 85)* |  |  |  |  |  |  |  |  |  |  |  |  |  |
| State anxiety (SAI) | 85 | 34.05 (6.45) | 29.28 (5.11) | −4.76 | [−5.87, −3.66] | 5.12 | −0.82 | [−1.07, −0.63] | −0.93 | −8.57 | 84 | < .001 | — |
| Mood | 81 | 6.95 (1.62) | 8.14 (1.29) | +1.19 | [0.93, 1.44] | 1.15 | +0.81 | [0.65, 1.00] | +1.03 | 9.26 | 80 | < .001 | < .001 |
| Stress | 77 | 4.60 (2.42) | 2.87 (2.00) | −1.73 | [−2.15, −1.31] | 1.85 | −0.78 | [−1.03, −0.59] | −0.94 | −8.21 | 76 | < .001 | < .001 |
| Clear-mindedness | 77 | 7.77 (1.90) | 8.09 (1.54) | +0.32 | [0.07, 0.58] | 1.11 | +0.19 | [0.04, 0.34] | +0.29 | 2.58 | 76 | .012 | .020 |
| Contentment | 77 | 7.08 (1.80) | 7.88 (1.69) | +0.81 | [0.43, 1.18] | 1.65 | +0.46 | [0.24, 0.70] | +0.49 | 4.27 | 76 | < .001 | < .001 |
| Calm | 75 | 6.97 (1.95) | 8.03 (1.57) | +1.05 | [0.59, 1.52] | 2.03 | +0.60 | [0.32, 0.91] | +0.52 | 4.49 | 74 | < .001 | < .001 |
| Restlessness | 75 | 3.59 (2.38) | 2.75 (2.15) | −0.84 | [−1.47, −0.21] | 2.75 | −0.37 | [−0.69, −0.10] | −0.31 | −2.65 | 74 | .010 | .020 |
| *Guided visit (n = 50)* |  |  |  |  |  |  |  |  |  |  |  |  |  |
| State anxiety (SAI) | 50 | 36.82 (8.48) | 29.96 (7.86) | −6.86 | [−9.18, −4.54] | 8.17 | −0.84 | [−1.27, −0.48] | −0.84 | −5.94 | 49 | < .001 | — |
| Mood | 50 | 7.12 (1.26) | 7.98 (1.70) | +0.86 | [0.38, 1.34] | 1.70 | +0.58 | [0.21, 1.07] | +0.51 | 3.57 | 49 | < .001 | .002 |
| Stress | 44 | 3.73 (2.03) | 2.36 (1.78) | −1.36 | [−1.98, −0.75] | 2.02 | −0.72 | [−1.14, −0.40] | −0.67 | −4.47 | 43 | < .001 | < .001 |
| Clear-mindedness | 44 | 7.89 (1.62) | 8.27 (1.60) | +0.39 | [−0.08, 0.86] | 1.54 | +0.24 | [−0.03, 0.57] | +0.25 | 1.66 | 43 | .104 | .104 |
| Contentment | 44 | 7.50 (1.47) | 8.39 (1.20) | +0.89 | [0.49, 1.28] | 1.30 | +0.66 | [0.40, 0.98] | +0.68 | 4.53 | 43 | < .001 | < .001 |
| Calm | 44 | 7.43 (1.45) | 8.18 (1.47) | +0.75 | [0.40, 1.10] | 1.16 | +0.51 | [0.27, 0.83] | +0.64 | 4.27 | 43 | < .001 | < .001 |
| Restlessness | 42 | 3.00 (2.06) | 2.21 (1.72) | −0.79 | [−1.28, −0.29] | 1.60 | −0.42 | [−0.75, −0.18] | −0.49 | −3.18 | 41 | .003 | .006 |
| *MAG (n = 64)* |  |  |  |  |  |  |  |  |  |  |  |  |  |
| State anxiety (SAI) | 64 | 33.78 (7.62) | 28.31 (7.02) | −5.47 | [−7.44, −3.50] | 7.89 | −0.75 | [−1.19, −0.41] | −0.69 | −5.54 | 63 | < .001 | — |
| Mood | 64 | 6.95 (1.37) | 8.17 (1.29) | +1.22 | [0.96, 1.48] | 1.03 | +0.91 | [0.72, 1.17] | +1.18 | 9.46 | 63 | < .001 | < .001 |
| Stress | 56 | 4.39 (2.26) | 2.70 (1.98) | −1.70 | [−2.17, −1.23] | 1.76 | −0.80 | [−1.12, −0.56] | −0.97 | −7.22 | 55 | < .001 | < .001 |
| Clear-mindedness | 56 | 7.91 (1.65) | 8.48 (1.19) | +0.57 | [0.21, 0.94] | 1.36 | +0.40 | [0.19, 0.63] | +0.42 | 3.14 | 55 | .003 | .005 |
| Contentment | 56 | 7.25 (1.67) | 8.29 (1.23) | +1.04 | [0.64, 1.43] | 1.46 | +0.72 | [0.49, 0.98] | +0.71 | 5.29 | 55 | < .001 | < .001 |
| Calm | 56 | 7.18 (1.61) | 8.29 (1.26) | +1.11 | [0.73, 1.49] | 1.42 | +0.77 | [0.53, 1.06] | +0.78 | 5.82 | 55 | < .001 | < .001 |
| Restlessness | 55 | 3.09 (2.19) | 2.42 (2.03) | −0.67 | [−1.29, −0.05] | 2.30 | −0.32 | [−0.66, −0.03] | −0.29 | −2.17 | 54 | .035 | .035 |
| *NHM (n = 71)* |  |  |  |  |  |  |  |  |  |  |  |  |  |
| State anxiety (SAI) | 71 | 36.24 (6.97) | 30.63 (5.27) | −5.61 | [−6.77, −4.44] | 4.91 | −0.92 | [−1.15, −0.73] | −1.14 | −9.62 | 70 | < .001 | — |
| Mood | 67 | 7.07 (1.60) | 7.99 (1.60) | +0.91 | [0.51, 1.31] | 1.66 | +0.57 | [0.27, 0.89] | +0.55 | 4.49 | 66 | < .001 | < .001 |
| Stress | 65 | 4.18 (2.37) | 2.68 (1.90) | −1.51 | [−2.01, −1.00] | 2.05 | −0.71 | [−0.99, −0.46] | −0.74 | −5.94 | 64 | < .001 | < .001 |
| Clear-mindedness | 65 | 7.72 (1.92) | 7.88 (1.78) | +0.15 | [−0.14, 0.45] | 1.18 | +0.08 | [−0.08, 0.24] | +0.13 | 1.06 | 64 | .295 | .295 |
| Contentment | 65 | 7.22 (1.74) | 7.88 (1.75) | +0.66 | [0.27, 1.05] | 1.57 | +0.38 | [0.14, 0.63] | +0.42 | 3.39 | 64 | .001 | .005 |
| Calm | 63 | 7.11 (1.94) | 7.90 (1.72) | +0.79 | [0.29, 1.30] | 2.02 | +0.43 | [0.15, 0.75] | +0.39 | 3.12 | 62 | .003 | .008 |
| Restlessness | 62 | 3.63 (2.34) | 2.68 (2.01) | −0.95 | [−1.58, −0.32] | 2.48 | −0.44 | [−0.77, −0.15] | −0.38 | −3.02 | 61 | .004 | .008 |

*Note.* MAG = Modern Art Gallery; NHM = Natural History Museum. SAI range: 20–80; VAS range: 1–10. n = participants with complete pre–post pairs; all statistics are computed on complete pairs. Δ = M post − M pre. d*_av_* = Δ divided by the average of the pre- and post-intervention standard deviations (Lakens, 2013); d*_z_* = Δ divided by the standard deviation of the change scores. Confidence intervals for d*_av_* are 95% percentile bootstrap intervals (3,000 resamples). p = two-tailed p value from paired-samples t tests; p Holm = p value adjusted within each group across the six VAS outcomes with the Holm procedure (not applied to the SAI, the confirmatory outcome). The MAG and NHM rows include both activities.
